# All-Cellulose Hydrogel-Based Bioinks for the Versatile 3D Bioprinting of Different Cell Lines

**DOI:** 10.1021/acs.biomac.4c01546

**Published:** 2025-02-04

**Authors:** João P. F. Carvalho, Nicole S. Lameirinhas, Maria C. Teixeira, Jorge L. Luís, Helena Oliveira, José M. Oliveira, Armando J. D. Silvestre, Carla Vilela, Carmen S. R. Freire

**Affiliations:** a CICECO−Aveiro Institute of Materials, Department of Chemistry, 56062 University of Aveiro, Aveiro 3810-193, Portugal; b CICECO−Aveiro Institute of Materials, EMaRT Group-Emerging Materials, Research, Technology, School of Design, Management and Production Technologies Northern Aveiro, Oliveira de Azeméis 3720-511, Portugal; c CESAM−Centre for Environmental and Marine Studies, Department of Biology, 56062 University of Aveiro, Aveiro 3810-193, Portugal

## Abstract

The development of bioink formulations with suitable properties is fundamental for the progress of 3D bioprinting. The potential of cellulose, the most abundant biopolymer, in this realm has often been underestimated, relegating it essentially to a reinforcement additive of bioinks. In this work, cell-laden bioink formulations, composed exclusively of cellulose, viz., “all-cellulose bioinks”, were developed by combining carboxymethyl cellulose (CMC) and nanofibrillated cellulose (NFC) in different mass proportions (90/10, 80/20, and 70/30%). The incorporation of NFC increases the printability of the inks (from Pr = 0.7 to 0.9) while maintaining their shear-thinning behavior, and increasing contents of NFC also decrease the degradation rate of the hydrogels after 7 days. The bioprinting of the cell-laden formulations, with HaCaT (keratinocyte) and ATDC5 (chondrogenic) cells, resulted in high (>80%) cell viabilities for up to 7 days, corroborating the versatility of the bioinks and their potential to originate distinct 3D living structures for biomedical applications.

## Graphical abstract

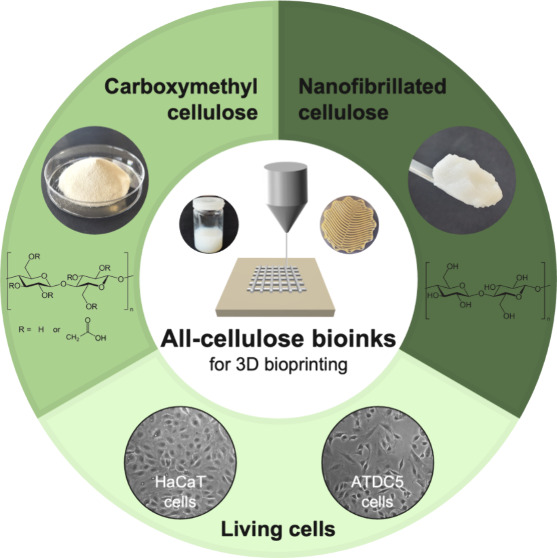

## 1 Introduction

Additive manufacturing has attracted the interest of multiple scientific and industrial sectors with the facile manufacturing of 3D objects in a precise computer-controlled way. This versatile technique, in which different materials are usually deposited layer-by-layer to achieve the bottom-up creation of complex structures, has been explored to build a plethora of different objects, including electronics,^1^ construction elements,^2^ foodstuffs,^3^ or even living tissues.^4^ In the latter case, a so-called “bioink” containing living cells (often combined with biomaterials) is used to originate complex 3D living structures that resemble native tissues.^5–7^ This branch of additive manufacturing technology is commonly referred to as 3D bioprinting. The potential of the 3D bioprinted living tissue analogs in biomedical areas is clear, since tissue engineering and regeneration and the development of drugs could be revolutionized by the *in vitro* tailored creation of tissue or tumor analogues.^8^ Nonetheless, the potential of 3D bioprinting is strongly dependent on the availability of bioinks with suitable rheological properties and biological performance, which have a major impact on the final performance of the 3D bioprinted living constructs.^6,9,10^

Multiple types of bioinks have been developed, but hydrogel-based options are the most explored for extrusion 3D bioprinting, given their remarkable properties, including rheological features, high water content, and resemblance to the cellular microenvironment.^11,12^ While hydrogels may be obtained from both natural and synthetic polymers, the use of naturally derived macromolecules is particularly relevant in the biomedical areas.^13^ Biopolymers, and specifically polysaccharides, are recognized by their intrinsic biocompatibility and low immunogenicity, and often straightforward cross-linking under mild conditions (*e.g*., exposure to divalent and trivalent cations).^14^ Indeed, multiple studies have explored the use of alginate, chitosan, and other polysaccharide-based hydrogels for 3D bioprinting endeavors.^15^

Cellulose, a polysaccharide composed of β(1→4) linked d-glucose units, present in plants, tunicates, and algae, is the most abundant polymer in nature.^16^ In the field of 3D bioprinting, cellulose nanoforms (i.e., cellulose nanocrystals, cellulose nanofibers, and bacterial nanocellulose) have found a common role as additives for the development of composite bioinks, with improved rheological properties and printability.^17,18^ Cellulose may alternatively be modified to produce different derivatives, namely cellulose esters or ethers, with a large diversity of properties depending on the imparted functionalities. In the specific case of carboxymethyl cellulose (CMC), the presence of carboxymethyl groups at C2, C3, or C6 increases the water solubility of this polysaccharide, allowing it to form viscous gel-like formulations that have found application in the cosmetic, pharmaceutical, and food sectors as excipients and thickening agents.^19^ Interestingly, CMC may be cross-linked with trivalent cations (e.g., Fe^3+^ and Al^3+^), originating stable hydrogels.^20^ However, the development of bioinks composed exclusively of cellulose (or its derivatives) remains underexplored. This is probably motivated by the assumption that the absence of adhesion motifs (e.g., arginylglycylaspartic acid (RGD) ligand) on cellulose will compromise the biological performance of cellulose-based bioinks.^21^ Nonetheless, several published works have shown the successful use of cellulose-based materials for biological endeavors, with high cell viabilities and cell proliferation, as appraised by Hickey and Pelling.^22^ In this context, the present work investigates the potential of cellulose in the realm of 3D bioprinting by designing all-cellulose hydrogel-based bioinks composed of CMC reinforced with NFC and loaded with living cells, namely HaCaT (keratinocyte) or ATDC5 (chondrogenic) cell lines. Although the combination of CMC and NFC has been described by Mohan et al. for the development of scaffolds via freeze-drying for posterior cell seeding,^23^ no previous work has explored the development of an all-cellulose hydrogel bioink of CMC and NFC for the 3D bioprinting of living cells, using ionic cross-linking, to originate living 3D structures from distinct cell lines. Herein, all-cellulose inks with different compositions were prepared and thoroughly characterized in order to evaluate the potential of these versatile bioink formulations for the 3D bioprinting of different cell types, originating 3D living structures that could be used for applications such as biomedical research and drug development.

## 2 Materials and Methods

### 2.1 Reagents and Materials

Phosphate-buffered saline (PBS, pH 7.4), iron­(III) chloride hexahydrate (>97%), and sodium carboxymethyl cellulose (CMC, *M*_w_: 700,000, DS: 0.80–0.95) were supplied by Sigma-Aldrich (Sintra, Portugal). Nanofibrillated cellulose (NFC) with fibrils with an average diameter of 20–50 nm and a carboxyl content of 0.14 ± 0.06 mmol g^–1^ was obtained from softwood bisulfite fibers via mechanical and enzymatic treatments and supplied by the VTT Technical Research Centre (Finland).^24^ Ultrapure water (type 1, 18.2 MΩ cm at 25 °C) was obtained using a Simplicity water purification system (Merck, Darmstadt, Germany).

Dulbecco’s modified Eagle’s medium (DMEM) for cell culture was purchased from PAN-Biotech (Germany). The trypsin-EDTA solution (0.25% trypsin, 0.02% EDTA), dimethyl sulfoxide (DMSO, ≥99.9%), and the LIVE/DEAD assay kit were obtained from Sigma-Aldrich (Sintra, Portugal), while 3-(4,5-dimethylthiazolyl-2)-2,5-diphenyltetrazolium bromide (MTT, 98%) was supplied by Alfa Aesar (Kandel, Germany). Penicillin/streptomycin (100×) and l-glutamine (200 mM) solutions were acquired from Grisp (Porto, Portugal). Fungizone and fetal bovine serum (FBS) were purchased from Gibco (Life Technologies, Carlsbad, CA, USA). The HaCaT cell line, an immortalized cell line of human keratinocytes, was obtained from Cell Lines Services (Eppelheim, Germany), and the ATDC5 chondrogenic cell line was kindly provided by Dr. Meriem Lamghari, from the i3s Instituto de Investigação e Inovação em Saúde, Universidade do Porto, Portugal.

### 2.2 Preparation of All-Cellulose Inks and of the Corresponding Hydrogels

The all-cellulose inks were formulated by the combination of CMC and NFC in different mass percentages (90/10, 80/20, and 70/30), for a final total solid content of 3.0 wt % in a total volume of 10.0 mL, as described in Table 1. NFC was previously thoroughly homogenized by mechanical stirring, ensuring the complete dispersion of NFC in the aqueous suspensions. All the inks were then extensively mixed mechanically to achieve the complete dissolution of CMC and ensure homogenization. The corresponding hydrogels were obtained by completely cross-linking the inks by immersion in an aqueous solution of FeCl_3_ at 1.0% (w/v) overnight.

**Table 1:** Composition of All-Cellulose Composite Inks

**sample**	**CMC [mg]**	**NFC [mg]**	**water [mL]**
CMC	300.0		10.0
CMC90:NFC10	270.0	30.0	10.0
CMC80:NFC20	240.0	60.0	10.0
CMC70:NFC30	210.0	90.0	10.0

### 2.3 Rheological Characterization

The rheologic properties of the inks and corresponding hydrogels were assessed using a Kinexus Lab+ rheometer (Malvern Instruments Limited, Malvern, United Kingdom) with a cone–plate geometry and a cone angle of 4°, at 20 °C. The rotational tests were performed with a 1 mm gap in a shear rate range of 0.1–100 s^–1^. The results were fitted to the power law model^25^ as depicted in eq 1

$$\eta =K\gamma^{\left(n-1\right)}$$ 1

in which η is the shear viscosity, *K* represents the consistency index, γ is the shear rate, and *n* corresponds to the flow index.

A three-step oscillatory test was used to evaluate the *G′* recovery rate (%) of the inks. Herein, the *G′* was first evaluated in relaxation, at 1 Pa for 1 min. Then, it was evaluated at 100 Pa for 10s. Finally, the *G′* in relaxation was assessed at 1 Pa for 1 min. The recovery rate (%) was calculated using eq 2:

$$\mathrm{recovery}(\%)=(G\prime_{\mathrm{recovered}}/G\prime_{\mathrm{initial}})\times 100$$ 2

in which *G′*_initial_ is the average *G′* in the first relaxation phase and *G′*_recovered_ is the *G′* in the last relaxation phase.

The viscoelastic evaluation of the hydrogels (*G′* and *G′′* moduli) was performed at a shear strain range of 0–100%, a 1 Hz frequency, and using cylinder-shaped samples of the cross-linked hydrogels (15 mm diameter × 5 mm height).

### 2.4 Mechanical Compression Assays

The mechanical properties of the prepared hydrogels were assessed through mechanical compression tests in a uniaxial Instron 5966 machine (Instron Corporation, Norwood, MA, USA) with a static load cell of 500 N, using cylindrical samples of the cross-linked hydrogels (15 mm diameter × 5 mm height) obtained as described in Section 2.2. The tests were executed in unconfined compression at a speed of 5 mm min^–1^ up to 80% strain, and the Young’s modulus was calculated with the Bluehill 3 software (version 3.22, Illinois Tool Works, Inc., Glenview, IL, USA).

### 2.5 Degradation Tests

The rate of degradation of the prepared hydrogels was evaluated in two different media (viz., DMEM and PBS) for up to 7 days at 37 °C. To achieve this, cylindrical samples (15 mm diameter × 5 mm height) were weighed and immersed in 2.0 mL of each medium. At defined time points, the samples were removed from the medium and weighed again after removal of the excess medium. The degradation rate was calculated using eq 3:

$$\mathrm{degradation}(\%)=[(W_{i}-W_{t})/W_{i}]\times 100$$ 3

where *W*(*i*) is the initial weight of the sample and *W*(*t*) is the corresponding sample weight at each time point.

### 2.6 Cell Culture and *In Vitro* Cytotoxicity Assessment

For all of the biological assays described in this work, cells were incubated at 37 °C in a 5% CO_2_ humidified atmosphere. HaCaT and ATDC5 cells were cultured with Dulbecco’s modified Eagle's medium (DMEM) supplemented with 2.0 mM l-glutamine, 250 μg mL^–1^ fungizone, 10% FBS, and 10000 U mL^–1^ penicillin/streptomycin and observed daily using an Eclipse TS100 microscope (Nikon, Tokyo, Japan). The hydrogels obtained by cross-linking of all-cellulose inks were incubated in culture medium for 24 h to prepare the corresponding extracts (30 mg mL^–1^), according to ISO10993-12.^26^

The assessment of the cytotoxicity of the all-cellulose inks was performed using the MTT assay.^27^ For each sample, six wells on a 96-well plate were seeded with living cells at specific cell densities: 6000 cells/well (for 24 h), 4000 cells/well (48 h), or 2000 cells/well (72 h). After 24 h, DMEM was replaced with 100 μL of the inks’ extracts and the plates were incubated for an exposure time of either 24, 48, or 72 h. Six wells were exposed only to plain culture media to act as controls for these experiments. After each exposure time, 50 μL of MTT (1.0 g L^–1^) was added to each well and left to incubate for 4 h. Posteriorly, the media were removed, 150 μL of DMSO was added to each well, and the plate was placed in an orbital shaker for 2 h. A BioTek Synergy HT plate reader (Synergy HT Multi-Mode, BioTeK, Winooski, VT) was used to measure the absorbance of each well at 570 nm, and the cell viability was calculated by applying eq 4:

$$\mathrm{cell}\;\mathrm{viability}(\%)=[({\mathrm{Abs}}_{\mathrm{sample}}-{\mathrm{Abs}}_{\mathrm{DMSO}})/({\mathrm{Abs}}_{\mathrm{control}}-{\mathrm{Abs}}_{\mathrm{DMSO}})]\times 100$$ 4

where Abs_sample_ corresponds to the absorbance of each well, Abs_DMSO_ to the absorbance of DMSO, and Abs_control_ to the absorbance of the controls.

### 2.7 3D Printing of All-Cellulose Bioinks

All the 3D printing experiments were performed using a 3D Bioplotter at 20 °C (Developer Series, Desktop Health, EnvisionTEC GMBH, Gladbeck, Germany). The optimization of the printing parameters (viz., printing speed and pressure) was performed by printing straight lines of 75 mm using a nozzle with a 0.41 mm inner diameter and varying pressure (1 to 2 bar) and speed (10 to 50 mm s^–1^). Considering the thickness and integrity of the filaments, a printing pressure of 1.5 bar and a speed of 30 mm s^–1^ were selected as appropriate parameters for 3D printing of the inks. Then, gridlike structures with 20 mm × 20 mm, a layer height of 0.320 mm, and a spacing of 2 mm were modeled using CAD software. These structures were printed using the prepared all-cellulose inks and immediately cross-linked by immersion in an aqueous solution of FeCl_3_ for at least 15 min.

The evaluation of the printability (Pr*)* of the all-cellulose inks was performed on 3D printed constructs using ImageJ software and by applying the following expression:

$$\mathrm{Pr}=L^{2}/16A$$ 5

in which *L* corresponds to the perimeter and *A* to the area of the square openings of the gridlike constructs.

### 2.8 Scanning Electron Microscopy (SEM)

Micrographs of the 3D printed constructs were acquired with an HR-FESEM SU-70 Hitachi microscope (Hitachi High-Technologies Corporation, Tokyo, Japan) at a 4 kV voltage. The samples were freeze-dried for 48 h, placed in an SEM stub with carbon adhesive tape, and coated using an EMITECH K950 carbon coating system before the SEM observations.

### 2.9 3D Bioprinting of HaCaT and ATDC5 Cells

The process of 3D bioprinting implies the inclusion of cells into the ink matrix. To achieve this, HaCaT or ATDC5 cells were incorporated into the CMC70:NFC30 ink. All samples were prepared following the methodology described in Section 2.2 in a sterile environment, and all reagents and materials were previously sterilized by at least 3 cycles of 20 min of UV irradiation. Cells were centrifuged and resuspended in 1 mL of DMEM and mixed with the formulations, originating bioinks with a final cell density of 2 × 10^6^ cells mL^–1^.

The 3D bioprinting of gridlike living structures was carried out as portrayed in Section 2.7. All of the 3D bioprinted living structures were incubated for up to 7 days (using the cell culture conditions described in Section 2.6). The cell viability was evaluated at defined time points (1, 3, or 7 days after the bioprinting procedure) using the LIVE/DEAD assay (EthD-1/calcein AM), following the specifications of the manufacturer. The structures were immersed in the fluorescent dies for 45 min at 37 °C and then observed using a confocal microscope (Zeiss LCM 880, Carl Zeiss, Oberkochen, Germany). The cell viability (%) was evaluated using eq 6:

cellviability(%)=[greenintensity/(red+greenintensity)]×100 6

### 2.10 Statistical Analysis

The analysis of variance (ANOVA) and Tukey’s test with statistical significance defined at *p* < 0.05 were performed using Origin software (OriginPro, version 9.0.0, OriginLab Corporation, Northampton, MA, USA). The analysis was always performed using at least three replicates, the corresponding mean values, and standard deviation.

## 3 Results and Discussion

The present study explores the development of bioinks obtained exclusively from cellulose (“all-cellulose bioinks”), by the combination of a cellulose derivative (CMC) with a cellulose nanoform, namely, nanofibrillated cellulose (NFC) and living cells (HaCaT or ATDC5) (Figure 1). Different ink formulations were prepared by combining various mass proportions of CMC and NFC (viz., 90/10, 80/20, and 70/30). An ink composed only of CMC (100/0) was also produced for comparison purposes. All samples were thoroughly characterized in terms of their rheologic behavior, namely, regarding their shear viscosity, shear stress, and *G′* recovery rate, and the corresponding cross-linked hydrogels were evaluated concerning their viscoelastic and mechanical properties, degradation rate in different media, and *in vitro* cytotoxicity against HaCaT and ATDC5 cells for up to 72 h. After the optimization of the extrusion parameters, the successful 3D bioprinting using cell-laden all-cellulose bioinks was performed and evaluated via LIVE/DEAD assays for different cell lines, aiming to understand the evolution of cell viability for up to 7 days after bioprinting and hinting at the potential of the living 3D structures for the biofabrication of living tissue analogues for distinct biomedical applications.

**Figure 1:**
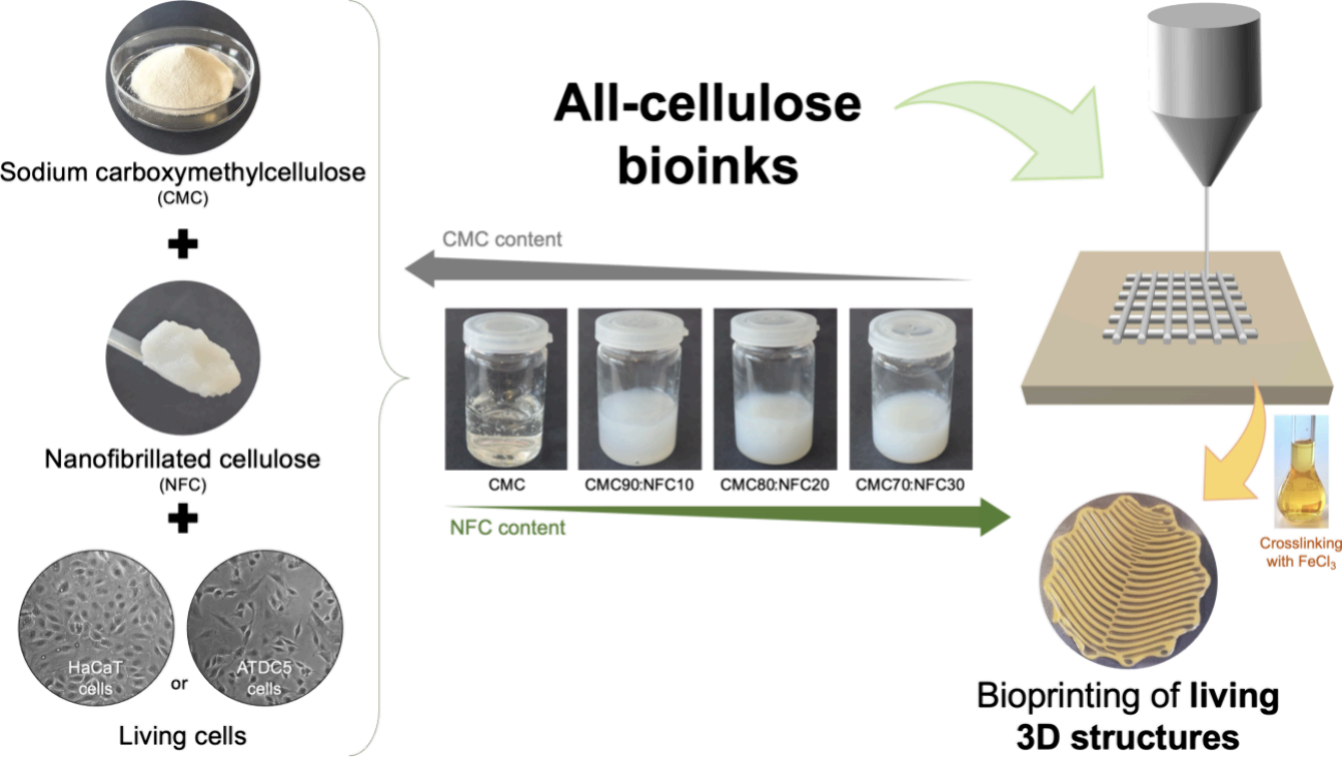
Schematic representation of the production of all-cellulose bioinks from sodium carboxymethyl cellulose, nanofibrillated cellulose, and living (HaCaT or ATDC5) cells for 3D bioprinting.

### 3.1 Characterization of the All-Cellulose Inks

The rheological properties of hydrogel-based inks play a pivotal role in the performance of this type of formulation in extrusion-based 3D bioprinting. Considering this, the all-cellulose ink formulations developed in this work were characterized in terms of their rheological profile (shear viscosity, shear stress, and *G′* recovery rate). As observed in Figure 2A, all samples exhibit a shear-thinning behavior, with a notorious decrease in shear viscosity with an increasing shear rate. This phenomenon, common for non-Newtonian fluids, is fundamental for extrusion 3D bioprinting, where the mechanical force applied will drive the passage of viscous materials through a nozzle.^10^ Understandably, the reduction of shear viscosity under stress allows an easier passage of the material through the small opening, avoiding clogging issues and protecting cells by reducing the intense shear stress associated with this process.^28^ Moreover, it was observed that the presence of NFC in the formulations does not greatly impact the shear viscosity or compromise the shear-thinning behavior of the inks, with all samples showing similar profiles to that of the CMC ink formulation. Similar results were reported, for instance, in the works of Markstedt et al., about the incorporation of NFC into an alginate hydrogel^29^ and of Lameirinhas et al.^30^ on the addition of NFC to a gellan gum hydrogel. On the other hand, the evaluation of the shear stress with an increasing shear rate (Figure 2B) reveals that CMC and CMC90:NFC10 samples have very similar behaviors, achieving values of 552 ± 24 and 545 ± 12 Pa, respectively, but higher NFC contents resulting in a lower shear stress at a high shear rate, with CMC80:NFC20 and CMC70:NFC30 presenting the lowest values, 495 ± 8 and 406 ± 6 Pa, respectively. As already stated, a reduction of the shear stress is very advantageous for the desired 3D bioprinting applications, where high shear stress could compromise the maintenance of elevated cell viabilities during the printing procedure.^31^ The fitting of these rheological results to the power law model^25^ confirms the adequate flow index for all samples (*n* < 1) and a high consistency index (*K >* 50), namely, 272.76 ± 2.44 for CMC and 235.26 ± 3.69 for CMC90:NFC10, with CMC:80:NFC 20 and CMC70:NFC30 showing values of 197.76 ± 1.93 and 190.58 ± 1.82, respectively, which is a behavior expected for non-Newtonian fluids.^31^

**Figure 2:**
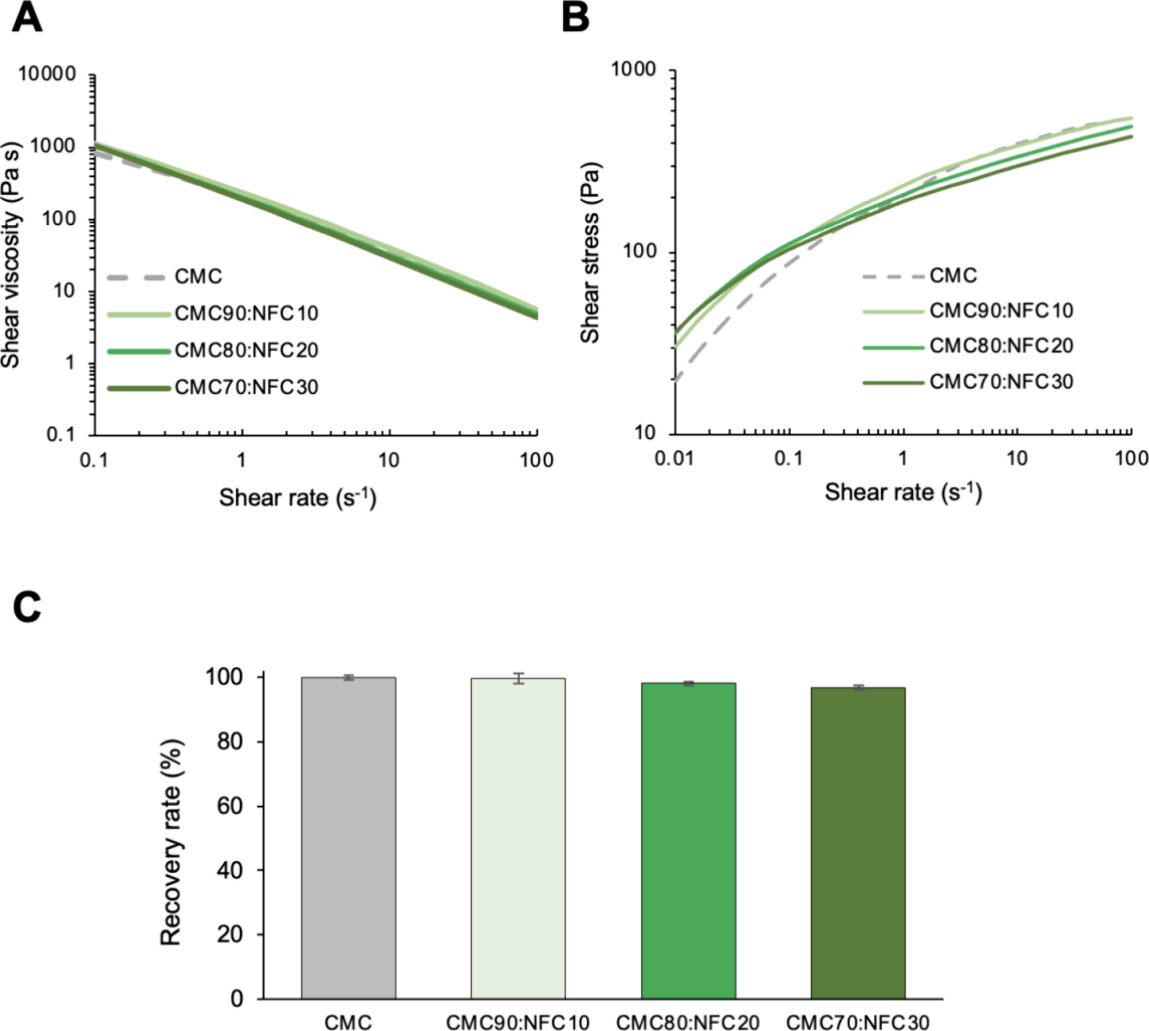
Rheological data of the all-cellulose inks: (A) shear viscosity as a function of the shear rate, (B) shear stress as a function of the shear rate, and (C) recovery rate (%) of the elastic modulus (*G′*). Values are presented as the mean of at least three replicates with no significant statistical differences.

Another parameter with high relevance for 3D bioprinting endeavors is the *G′* recovery rate (%). Herein, the elastic modulus (*G′*) of the ink was measured before and after a period of intense stress, mimicking the forces applied in the extrusion printing process. Inks with high recovery rates (>80%) can recover nearly all of their viscoelastic properties when deposited in the printing platform, right after the passage through the nozzle, a feature that relates to their capacity of maintaining the integrity of the predesigned structure.^10,31^ The data shown in Figure 2C show that all samples maintain very high recovery rates, with values of 99.89 ± 0.61% (CMC), 99.58 ± 1.56% (CMC90:NFC10), 98.02 ± 0.48% (CMC80:NFC20), and 96.83 ± 0.72% (CMC70:NFC30), thus confirming that all formulations are able to recover nearly all their rheological properties after intense stress and that the addition of NFC does not compromise this behavior. Furthermore, all these values are above the threshold of 85% suggested by Kiyotake et al. for hydrogel-based bioinks for 3D bioprinting.^32^ Hernandez-Sosa and colleagues have very recently described the same behavior in alginate hydrogels reinforced with NFC, with no major change of the recovery rate, which remained around 84%,^33^ a value that is below the ones obtained for the all-cellulose bioinks developed in this work.

As expected, the addition of NFC to the formulations also modifies the macroscopic aspect of the inks, as observed in Figure 1, with NFC-containing samples demonstrating the pearly white color characteristic of the NFC suspension and therefore being very different from the translucent CMC ink.

### 3.2 Characterization of the Cross-Linked Hydrogels

The hydrogels obtained by the cross-linking of the all-cellulose inks were characterized regarding their viscoelastic and mechanical properties, degradability in different media, and *in vitro* cytotoxicity against different cell lines.

The evaluation of the elastic (*G′*) and viscous (*G′′*) moduli of the samples, shown in Figure 3A, reveals that *G′* is higher than *G′′* for all samples, proving that the hydrogels exhibit a solid-like nature at the viscoelastic region that allows them to maintain their shape.^34^ This confirms the success of the cross-linking of the all-cellulose ink formulations with FeCl_3_, which occurs due to the interactions between Fe^3+^ ions and the carboxylic groups of CMC, resulting in hydrogels with solid-like behavior and high stiffness,^35–37^ and corroborates their use for the development of stable constructs that should keep the predesigned shape after printing.^38^ Moreover, these results agree with the works of Han et al. about alginate/gelatin/NFC bioinks^39^ and Radeke et al. on hydrogels from carboxymethylated nanocellulose and ECM proteins, where all hydrogels have also shown *G′* > *G′′.* ^40^

**Figure 3:**
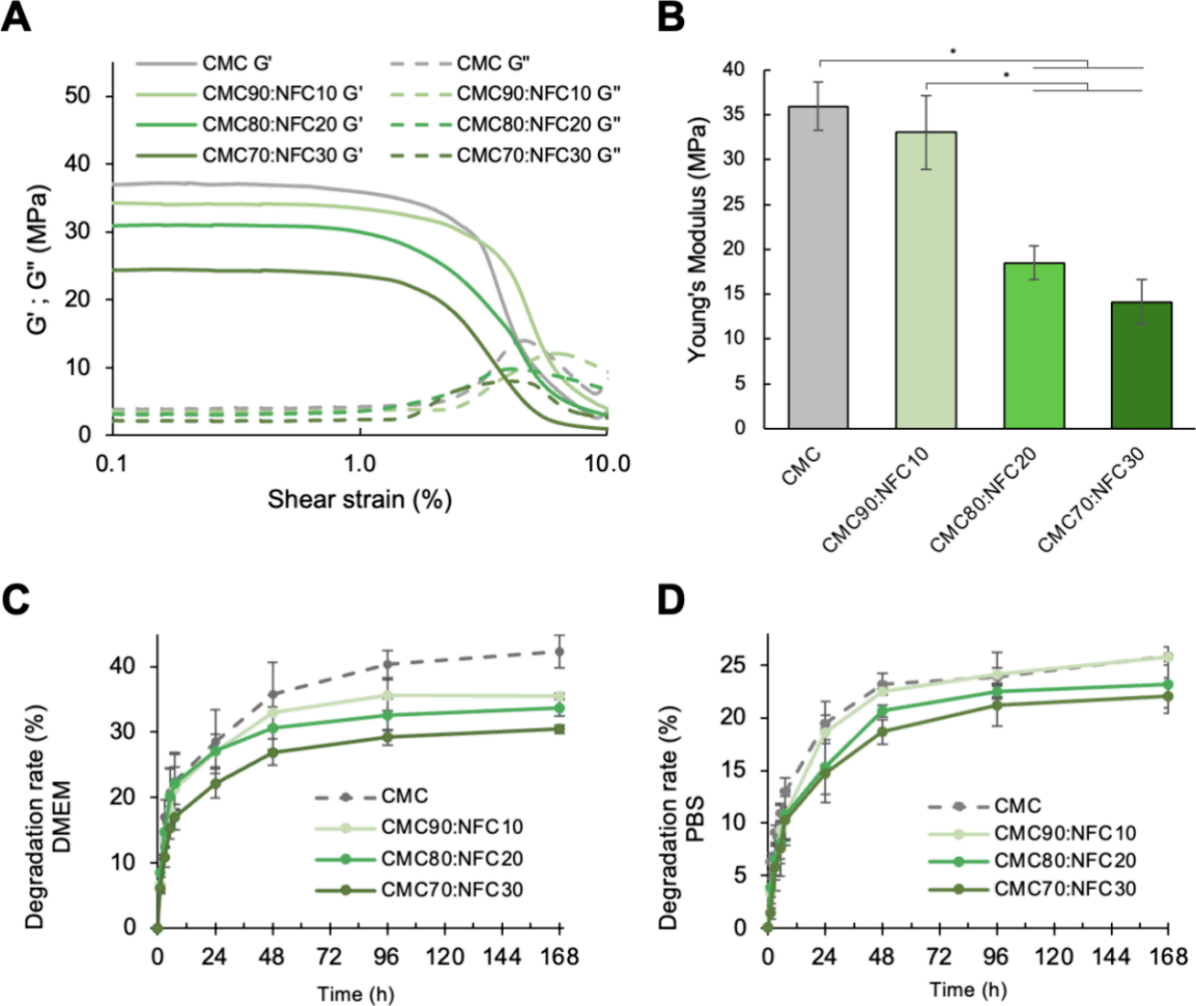
(A) Viscoelastic evaluation of the *G′* and *G′′* of the hydrogels obtained from all-cellulose inks, (B) Young’s modulus of the all-cellulose inks, and (C,D) degradation rate (%) of the all-cellulose inks in DMEM (C) and in PBS (D) for up to 7 days at 37 °C. Presented values are the mean of at least 5 replicates (*p < 0.05).

Regarding the mechanical properties of these hydrogels, the results of the compression assays (depicted in Figure 3B) demonstrate that the CMC sample possesses a Young’s modulus of 35.92 ± 2.68 MPa. Such a value is indicative of a very rigid hydrogel, which could affect the cell phenotype, proliferation, and differentiation.^41^ Interestingly, an increase in the NFC content of the inks significantly reduces the Young’s modulus of the samples, with CMC90:NFC10 showing 33.04 ± 4.09 MPa and samples CMC80:NFC20 and CMC70:NFC30 showing a very significant reduction, with 18.51 ± 1.91 and 14.14 ± 2.46 MPa, respectively, achieving values similar to certain biological tissues (viz., cartilage),^42^ and these values are higher than those obtained by Antich et al.^43^ with hyaluronic acid/alginate bioinks (around 3 MPa) and Mouser et al.^44^ for methacrylated hyaluronic acid-based hydrogels (around 8 MPa), both for cartilage bioprinting. Overall, the results obtained in this study reveal that the all-cellulose hydrogels developed in this work are less rigid than those of the pure CMC counterpart. This decrease is probably related with the fact that the lower CMC content in the formulations leads to a less extensive cross-linking and the eventual interference of NFC in the stiffness of the hydrogel, which could be used to fine-tune the rigidity of the hydrogels.

When submerged in culture media (DMEM) for 7 days at 37 °C, mimicking the cell culture conditions, the CMC hydrogels show a final degradation rate of 42.31 ± 2.54%, while all NFC-containing formulations show lower values (CMC90:NFC10 with 35.44 ± 0.51%, CMC80:NFC20 with 33.79 ± 1.34%, and CMC70:NFC30 with 30.47 ± 0.63%), as shown in Figure 3C. Therefore, the presence of NFC reduced the degradation rate of the cross-linked hydrogels. This is justified by the intrinsic characteristics of NFC, including its high chemical stability and crystallinity.^45^ A similar behavior was observed for the stability test performed using phosphate-buffered saline (PBS) at pH 7.4, an analogue of biological fluids, with all-cellulose formulations revealing a degradation lower than that of CMC (Figure 3D). However, a lower value range was seen for the test performed in PBS, probably due to the higher complexity of the composition of cell culture media, when compared to the simple saline buffer.^46^ Interestingly, these degradation rates are below the ones described by Mohan et al. for composite inks of CMC (6 wt %) and NFC (1.5 wt %), where a degradation rate of nearly 50% is reported after 7 days in culture media at 37 °C for the freeze-dried cellulose-based scaffolds, probably justified by the absence of ionic cross-linking.^23^

When developing new ink formulations for 3D bioprinting, their noncytotoxic nature is a very important characteristic since one of the main goals of the procedure is to create platforms where cells will be able to survive and thrive. Therefore, the cytotoxic effect of the prepared hydrogels was evaluated against HaCaT (keratinocyte) and ATDC5 (chondrogenic) cells. As shown in Figure 4, all samples are noncytotoxic against these cell lines for up to 72 h, with cell viabilities always above the 70% threshold defined by the ISO 10993-5:2009, a standard for the evaluation of the cytotoxicity of biomaterials.^47^ Specifically, the cell viability of HaCaT cells (Figure 4A) was maintained for up to 72 h of exposure to all samples, viz., 97.7 ± 9.3% (CMC), 100.6 ± 8.1% (CMC90:NFC10), 99.1 ± 7.1% (CMC80:NFC20), and 97.6 ± 10.1% (CMC70:NFC30). ATDC5 cells demonstrated a similar behavior, with 99.1 ± 8.5% for CMC, 99.1 ± 8.1% for CMC90:NFC10, and 99.9 ± 2.2 and 97.9 ± 8.8% for CMC80:NFC20 and CMC70:NFC30, respectively, as seen in Figure 4B. This is in line with the known cytocompatibility of both NFC^48^ and CMC,^19^ and similar outcomes have been described by multiple research groups with different cell types for hydrogels using NFC in their composition, together with biopolymers like cellulose,^23^ alginate,^29,49^ and gellan gum.^30^ These results indicate the safety of the use of all-cellulose inks against different cell lines and thus confirm their potential to be laden with cells and used in 3D bioprinting applications.

**Figure 4:**
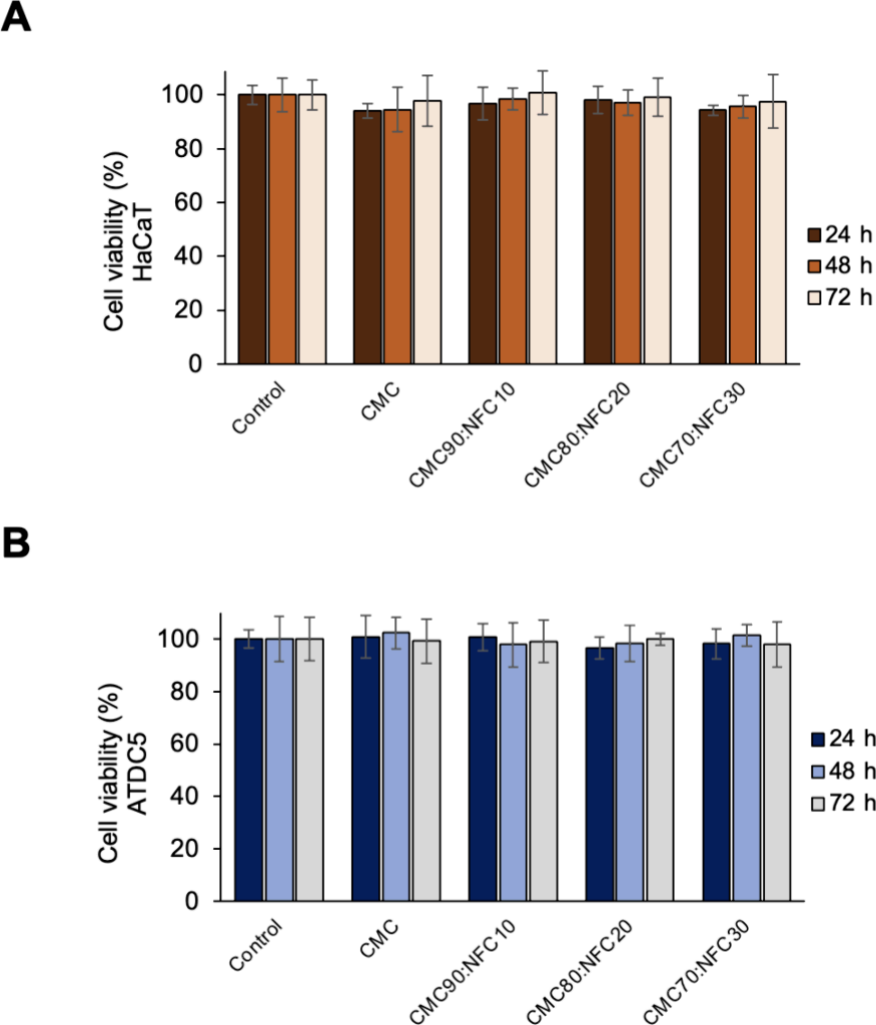
Evaluation of the cell viability (%) of HaCaT (A) and ATDC5 (B) cells after 24, 48, or 72 h of exposure to the CMC:NFC inks. Shown values are the mean of at least six replicates, with no significant difference from the respective controls.

### 3.3 3D Printing Using All-Cellulose Inks

The optimization of the extrusion 3D bioprinting parameters is fundamental to achieve an adequate balance between printing pressure and printing speed, allowing the dispensing of adequate quantities of material without compromising the resolution of the constructs and cell viability. Herein, this optimization process (exemplified in Figure 5A for the CMC70:NFC30 formulation) revealed that the all-cellulose inks may be successfully printed using a nozzle with a 0.41 mm inner diameter, a printing speed of 30 mm s^–1^, and a printing pressure of 1.5 bar. These conditions were chosen by considering the width and the integrity of the printed straight filaments. Given so, gridlike structures with multiple layers were 3D printed using the optimized printing conditions with all the developed hydrogel formulations, namely, CMC, CMC90:NFC10, CMC80:NFC20, and CMC70:NFC30.

**Figure 5:**
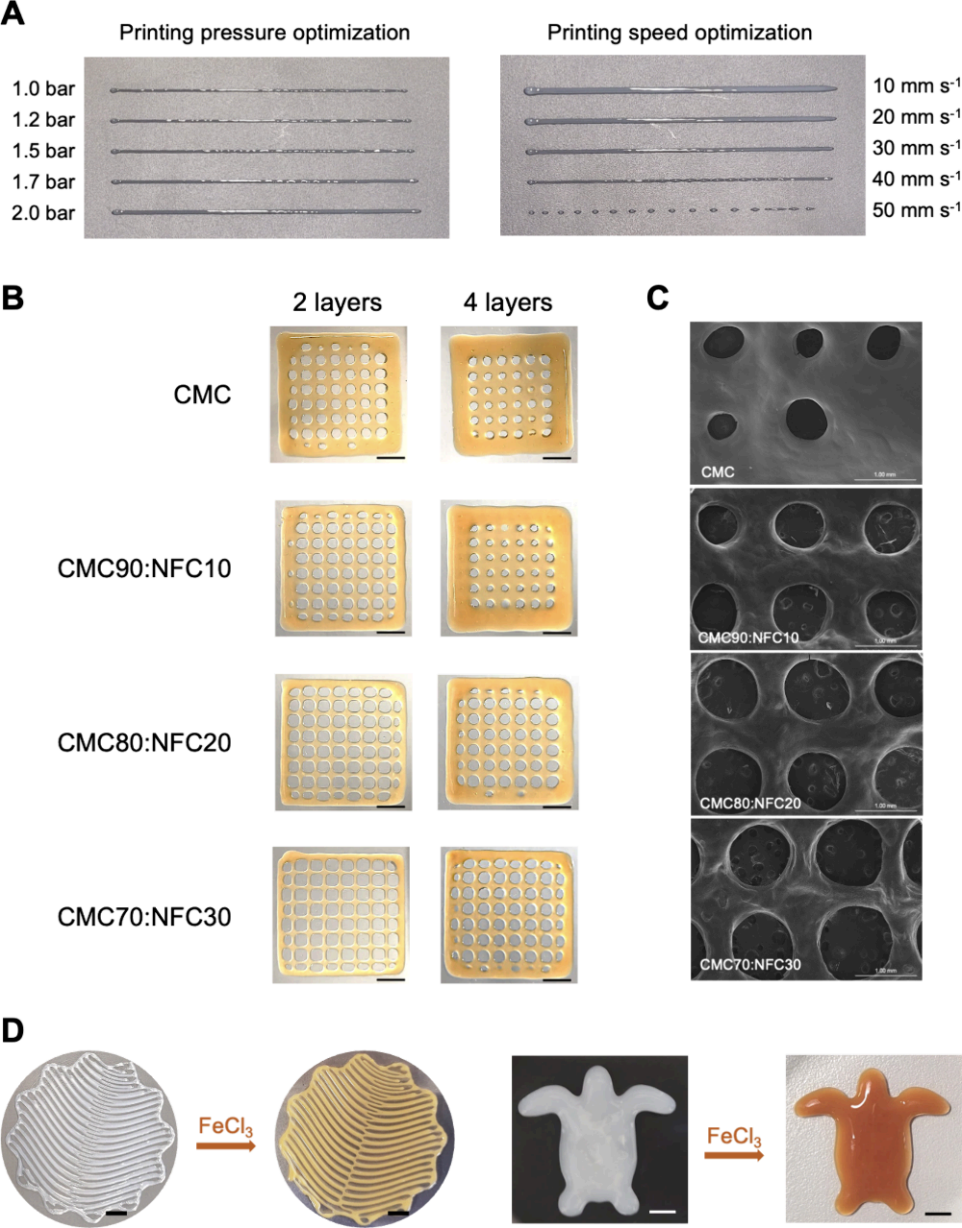
3D printing assays using the all-cellulose inks: (A) photographs of the 3D printed straight filaments for the optimization of printing pressure and printing speed, exemplified for CMC70:NFC30; (B) 3D printed gridlike structures with multiple layers. Scale bars represent 5.0 mm; (C) SEM micrographs of 3D printed gridlike structures obtained using the CMC, CMC90:NFC10, CMC80:NFC20, and CMC70:NFC30 formulations; (D) 3D printed two-layered intricate structure and nine-layered turtle shape (6.0 mm height) using CMC70:NFC30 before and after the cross-linking with FeCl_3_. Scale bars represent 5.0 mm.

As observed in Figure 5B, the increasing content of NFC results in 3D structures with an enhanced resolution, with the gridlike structures showing a better definition of the square pores between filaments. This was further confirmed by the SEM observation of the structures, shown in Figure 5C, where the difference between the pores of the CMC structure and those of the NFC-containing counterparts can be clearly perceived. Nonetheless, this was additionally assessed by the evaluation of the printability (Pr), which has shown that CMC (Pr = 0.7) and CMC90:NFC10 (Pr = 0.8) have values below the desired printability for 3D bioprinting, which should be in the range of 0.9–1.^10^ Only the formulations with higher NFC contents, namely, CMC80:NFC20 and CMC70:NFC30, fulfilled this requirement, with a Pr = 0.9. Other bioinks based on polysaccharides have shown similar printability results, including the work of Teixeira et al.^46^ where the addition of lysozyme nanofibrils increased the printability of alginate hydrogels (to Pr = 0.9) and the work of Butler et al.^50^ with starch and chitosan-based bioinks and Davern et al.^51^ with gelatin methacryloyl bioinks with Laponite. The superior printability of the CMC70:NFC30 formulation was also confirmed by 3D printing more intricate and complex structures, as seen in Figure 5D, which also show that the pearly white colored structures clearly acquired the typical orange coloration of the FeCl_3_ solution.

### 3.4 3D Bioprinting of HaCaT or ATDC5-Laden All-Cellulose Bioinks

The aim of this work was to develop new all-cellulose bioinks for 3D bioprinting containing living cells and to successfully originate 3D living structures, which may find application in the biomedical field. Given this, bioinks were developed by the combination of the CMC70:NFC30 formulation with different cell lines, in order to prove its versatility. This formulation was chosen considering not only its enhanced characteristics, including rheological properties and printability but also the mechanical properties and improved stability of the corresponding hydrogels in different media. The results of the LIVE/DEAD assays performed 1, 3, and 7 days after the 3D bioprinting procedure, as depicted in Figure 6A, followed the standard procedure employed in multiple studies to access cell viability in the area of bioinks for 3D bioprinting,^18,30,32,36,46,52,53^ showing that living HaCaT cells (marked in green) are homogeneously spread throughout the matrix, with a clear prevalence of living cells over dead cells (marked in red). In fact, the cell viability is very high on the first day after bioprinting (82 ± 4%) and remains elevated not only after 3 days (84 ± 3%) but up to 7 days (88 ± 1%), as illustrated in Figure 6B. Similarly, ATDC5 cells were evenly distributed along the constructs obtained from the cell-laden CMC70:NFC30 bioink, and the number of alive cells clearly surpasses the number of dead ones. The ATDC5 cell viability was 81 ± 4% after 1 day, later achieving values of 86 ± 4% after 3 days and 89 ± 2% at 7 days postbioprinting. These results are very similar to the ones reported in a different work using NFC-containing bioinks for the 3D bioprinting of HaCaT cells, where cell viabilities of 90 ± 3% after 7 days were observed.^30^ Interestingly, the work of Lan and colleagues with TEMPO-oxidized NFC and alginate for the 3D bioprinting of fibrochondrocytes showed lower cell viabilities (around 70%) after the same period.^54^ Kim et al., who used different biopolymeric hydrogels for the 3D bioprinting of ATDC5 cells, also describe cell viabilities of around 80% after 7 days.^55^ Similar cell viability results have also been observed in works using different polysaccharide-based hydrogels for the 3D bioprinting, including pectin,^52,56^ gellan gum,^30,57^ chitosan,^58,59^ and alginate.^18,60^ Altogether, the results corroborate the potential of the CMC70:NFC30 formulation to be loaded with different types of cell lines and to be used in 3D bioprinting endeavors with high biological performance, hinting at the potential of these bioinks and of cellulose itself to be used for the biofabrication of *in vitro* tissue analogues.

**Figure 6:**
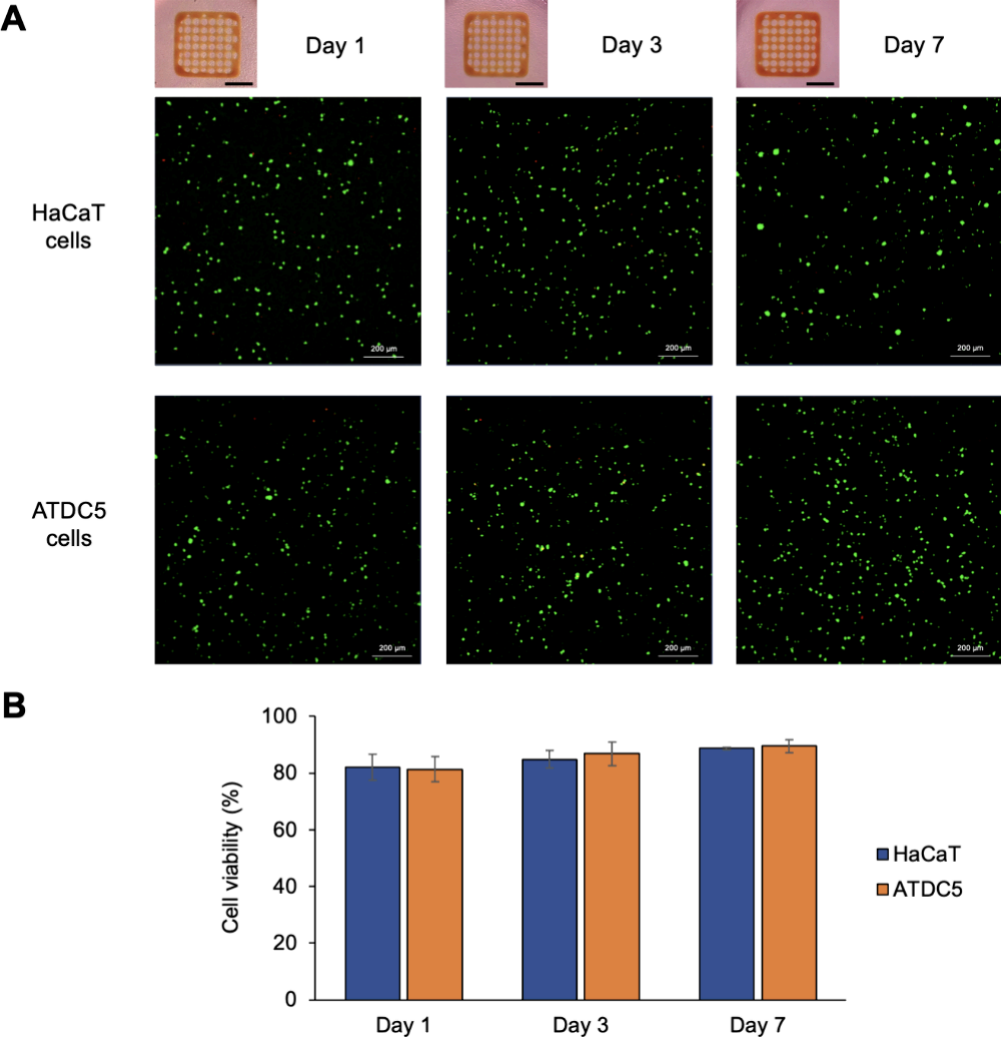
Cell viability on 3D bioprinted structures using the CMC70:NFC30 bioinks (A) LIVE/DEAD fluorescence micrographs of HaCaT and ATDC5 cells in the 3D bioprinted gridlike structures 1, 3, and 7 days after bioprinting. Scale bars represent 10.0 mm; (B) determination of HaCaT and ATDC5 cell viability (%) 1, 3, and 7 days postbioprinting. The data presented here correspond to the mean of at least three replicates.

## 4 Conclusions

The present work explores the development of bioinks based exclusively on the use of cellulose, i.e., “all-cellulose bioinks”, using a water-soluble cellulose derivative, carboxymethylcellulose, and a cellulose nanoform, nanofibrillated cellulose, for the 3D bioprinting of different cell lines. The thorough characterization of the formulations revealed their good rheological properties, with notorious shear-thinning behavior and *G′* recovery rates, and the presence of NFC increasing their printability while also providing additional stability to the ionically cross-linked hydrogels in different media. Moreover, the use of different contents of NFC modified the mechanical properties of the final constructs, mimicking the stiffness of biological tissues. After the optimization of the extrusion printing parameters, the successful 3D bioprinting of living tissue analogues with the CMC70:NFC30 formulation loaded with either HaCaT (skin) or ATDC5 (cartilage) cells resulted in very high viabilities (above 80%) up to 7 days postbioprinting for both types of cells. These results confirm the potential and versatility of these formulations for the extrusion-based 3D bioprinting of living cells, originating 3D living structures that could find application in important biomedical areas like tissue engineering and drug testing, further corroborating the potential of cellulose to be used as a material for these endeavors. Future studies on the use of these 3D structures for biomedical applications could explore other specific biological parameters, like cell proliferation, functional behavior, or cell–cell and cell–matrix interactions at different and longer periods, in order to further investigate the biological potential of the bioinks developed here.

## Abbreviations

CMC: carboxymethyl cellulose
DMEM: Dulbecco’s modified Eagle’s medium
DMSO: dimethyl sulfoxide
EDTA: ethylenediaminetetraacetic acid
FBS: fetal bovine serum
MTT: 3-(4,5-dimethylthiazolyl-2)-2,5-diphenyltetrazolium bromide
NFC: nanofibrillated cellulose
PBS: phosphate-buffered saline
RGD: arginylglycylaspartic acid
TEMPO: 2,2,6,6-tetramethylpiperidine-1-oxyl
UV: ultraviolet
